# In vitro surface characteristics and impurity analysis of five different commercially available dental zirconia implants

**DOI:** 10.1186/s40729-018-0124-8

**Published:** 2018-04-26

**Authors:** B. Beger, H. Goetz, M. Morlock, E. Schiegnitz, B. Al-Nawas

**Affiliations:** 1 Department of Maxillofacial Surgery, University Medical Center of the Johannes Gutenberg-University Mainz, Augustusplatz 2, 55131 Mainz, Germany; 2 Biomaterials in Medicine (BioAPP), University Medical Center of the Johannes Gutenberg-University Mainz, Mainz, Germany

**Keywords:** Zirconia, Dental implant, Ceramic, Implant surface, Implant material, Roughness, Titanium

## Abstract

**Background:**

The aim of this study was to assess surface characteristics, element composition, and surface roughness of five different commercially available dental zirconia implants.

Five zirconia implants (Bredent whiteSKY™ (I1), Straumann® PURE Ceramic (I2), ceramic.implant vitaclinical (I3), Zeramex® (I4), Ceralog Monobloc M10 (I5)) were evaluated.

**Methods:**

The evaluation was performed by means of scanning electron microscopy (SEM), energy-dispersive X-ray spectroscopy (EDX), and confocal laser scanning microscopy (CLSM).

**Results:**

The semi-quantitative element composition showed no significant impurity of any implant tested. Both the machined and the rough areas of the investigated implants were predominated by zirconium, oxygen, and carbon. Roughness values (*S*_a_) showed highest values for I2 and I5.

**Conclusions:**

The investigated zirconia implants showed surface characteristics and roughness values close to those of conventionally produced titanium implants, making them a promising alternative. However, zirconia implants have yet to prove themselves in clinical practice and clinical controlled trials.

## Background

Dental implants have become a well-established treatment method for oral rehabilitation after tooth loss. Pure titanium is still the material of choice when it comes to dental intraosseous implants and has been used for decades. However, titanium implants have esthetic limitations, especially in the front aspect of the maxillary jaw. The recession of the gingiva can lead to visible implant necks. Furthermore, titanium may cause immunological reactions with early local infection and possible risk for implant loss [1]. Ceramic implants are proclaimed as a new alternative to titanium implants. The first tooth-colored ceramic implants were inferior to titanium-based implants due to their biomechanical characteristics such as low fracture toughness [2]. In the 1980s, the Tübinger immediate implant was introduced, fully made of aluminum oxide (AL_2_O_3_), but was withdrawn from the market because of high fracture rates [3]. Other investigations on different AL_2_O_3_ implants found less bone-implant contact compared to titanium [4] as well as reduced survival rates [2, 5]. Since the introduction of yttrium-stabilized tetragonal zirconia polycrystalline (Y-TZP)-based implants, it could be shown that these implants show high similarity in osseointegration compared to titanium implants [2].

Titanium implants with smooth or roughened surfaces have shown high success rates in various indications [2, 6, 7]. Surface characteristics of dental implants, as a new development over the last decades, are seen as an important factor that affects osseointegration, especially in compromised patients (e.g., following radiation therapy, bone augmentation, class D4 bone) [8]. By improving the implant design, implant material, and implant surface characteristics as well as surgical techniques and implant loading conditions, osseointegration can be affected [9]. Several new techniques are performed nowadays to speed up the osseointegration process by altering the surface of the implant chemically (incorporating inorganic phases onto the titanium oxide layer) or physically (increasing the level of roughness) [10, 11]. Advantages of surface-modified implants include (a) establishing a greater contact area followed by better primary stability, (b) providing surface-retaining blood clots, and (c) stimulating bone formation [10, 12]. In vitro tests of surface roughness showed higher proliferation, cytokine, and growth factor production of osteoblast-like cells. Those factors are known to affect proliferation, differentiation, and matrix synthesis of chondrocytes [13–16]. Many studies on surface characteristics of titanium implants were performed over the last years. Due to the renaissance and new development of zirconia implants, it is now necessary to study their behavior and surface characteristics and to compare them to titanium implants. However, data regarding the surface characteristics of these zirconia implants are very rare. Therefore, the aim of this study was to examine the surface characteristics, element composition, and surface roughness of the five different commercially available dental zirconia implants.

## Methods

### Investigated implants

The following five commercially available dental zirconia implants were used in this study (Table 1). Bredent whiteSKY™ implant (I1) is made from unground Brezirkon™, an yttrium oxide (Y_2_O_3_)-stabilized tetragonal polycrystalline zirconium oxide and is sandblasted. Zirconium oxide is endowed with 3 mol% yttrium oxide to gain a rectangle and room temperature stable structure [17]. Straumann® PURE Ceramic Implant (I2) is generally made from yttrium oxide-stabilized tetragonal polycrystalline zirconium oxide. The surface due to the manufacturer is coated with a special process called ZLA™ which shall be similar to the SLA™ process (Sandblasted, Large-grit, Acid-etched) of titanium implants. Ceramic.implant vitaclinical (I3) is made from zirconium oxide. The Zeramex® implant (I4) is made from zirconium and has a sandblasted and etched surface structure with their so-called ZERAFIL™ technology. Camlog’s Ceralog Monobloc M10 ceramic implant (I5) is also made from yttrium-stabilized zirconium dioxide. Unlike the other ceramic implants, it is produced with ceramic injection molding (CIM) technique. This technique requires no sandblasting or etching. The implants’ geometrical design and the surface structure are already molded via CIM before the sintering and hot isostatic pressing (HIP) process.

**Table 1:**
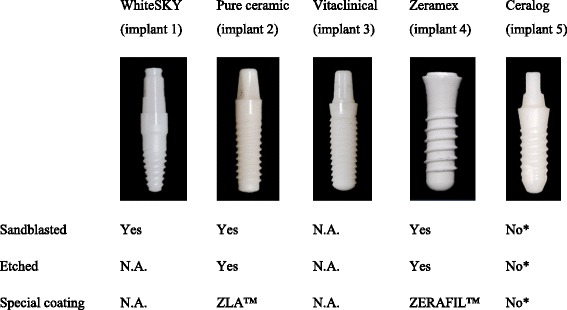
Five commercially available ceramic implants and surface characteristics

*Due to the processes CIM and HIP, see the “Methods” section

### Scanning electron microscopy

**Fig. 1:**
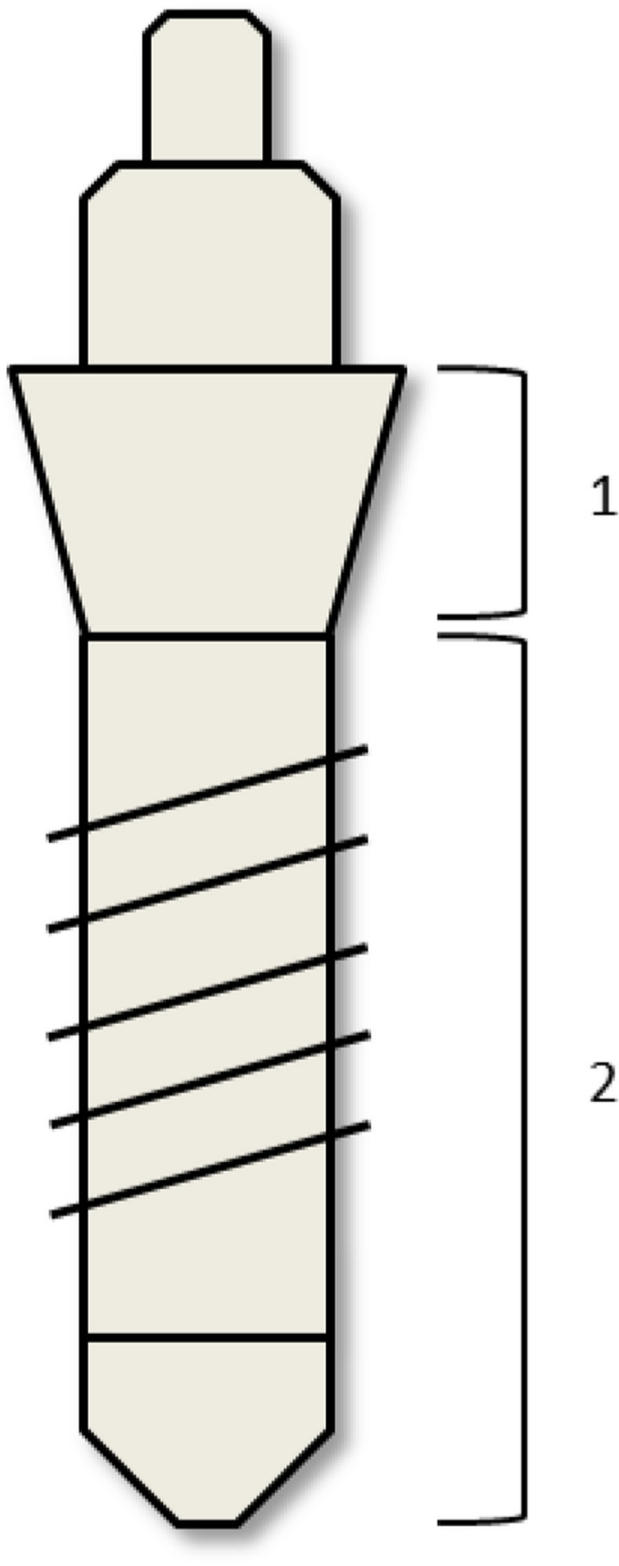
Diagram of different implant areas used for sampling. 1) Machined (untreated) area. 2) rough (treated) area

For a more detailed illustration of the implant surface topology, the technique of scanning electron microscopy (SEM) was used. A Quanta 200 FEG (FEI Company, Netherlands) field emission SEM equipped with environmental low vacuum mode makes it possible to avoid the typical surface charging-up problems of uncoated highly insulating ceramic implants without the need for sample preparation. Therefore, high-resolution SEM images with magnifications up to 25,000 are possible to demonstrate the micro-structured appearances at different locations. Comparable areas for all implants under investigation are selected by splitting up the cylindrical shape of the implant into sections (Fig. 1). For the comparison of surface structures between the tested implants, two regions of interest were selected: machined and rough area (compare Figs. 1 and 3). Each section was observed under different degrees of magnifications (× 2000, × 10,000, × 25,000) with the same microscope parameters (HV 20 kV, Det LFD, pressure 0.90 mbar). The low vacuum pressure in the sample chamber was reduced until charging levels on the sample surface were reduced to the level at which electron imaging of the sample surface was possible.

### Energy-dispersive X-ray spectroscopy

**Fig. 2:**
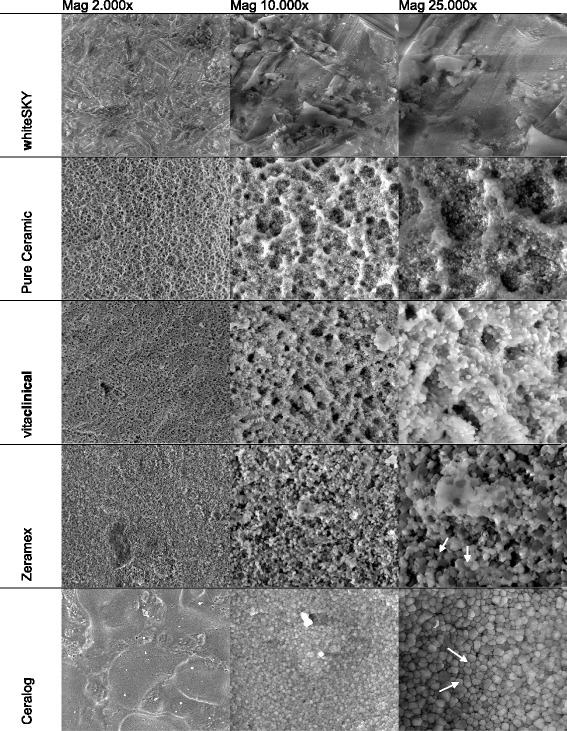
SEM. White arrow (→) exemplary mark the droplet like shape of surface as described in the text

**Fig. 3:**
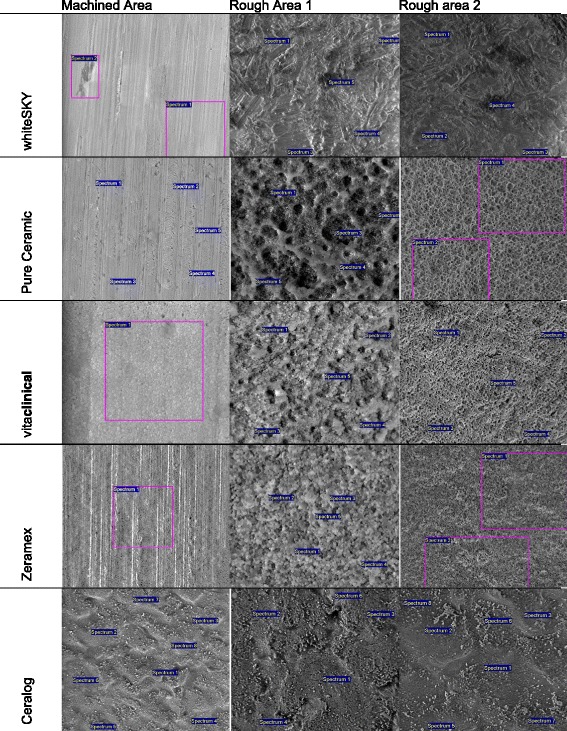
SEM for localization of EDX analysis

Analysis of the element composition of the implant surfaces by means of energy-dispersive X-ray spectroscopy (EDX) was performed with an INCA Energy 350 system (Oxford Instruments, Wiesbaden, Germany) coupled with the SEM Quanta 200 FEG (Fig. 2). Similar to the micro-morphological presentation, each implant was divided into comparable sites of interest. Typical areas were selected and evaluated (Fig. 3). With the “Point &ID” mode of the INCA Energy software, both points of interest and the areas of interest are selected for the EDX analysis. Microscopic conditions (magnification × 2000) and excitation energy (HV 20 kV) are kept constant for all types of implants. For a semi-quantitative approach, the main components identified on all of the sample surfaces are evaluated as shown in Table 2. Intervals of minimum and maximum values are presented to demonstrate the high inhomogeneous situation found at most of the selected areas.

**Table 2:** EDX

Element composition/semi-quantitative evaluation								
Location	Type	Zr at %_min_–at %_max_	Hf at %	Y at %_min_–at %_max_	Al at %_min_–at %_max_	O at %_min_–at %_max_	C at %_min_–at %_max_	N at %_min_–at %_max_
Machined area	WhiteSKY	16.0–19.5	< 0.25	1.47–1.67	< 0.5	55–58	17.6–24.0	< 1.0
	Straumann ZLA	19.4–22.4	< 0.35	1.6–1.8	< 0.12	48.5–52.1	20.6–22.3	5.2–7.2
	Vitaclinical	23.7	< 0.30	< 1.5	< 0.13	56	9.8	None
	ZERAMEX	17.7	< 0.23	< Det. limit	< 9.6	57.5	7.7	7.7
	Monobloc M10	4.0–11.0	< 0.09	< Det. limit	0.4–2.3	12.0–21.0	63.0–80.0	0–11.0
Rough area	WhiteSKY	15.6–19.3	< 0.23	0–2.8	1.1–3.8	49.8–80.7	0–20.7	0–6.3
	Straumann ZLA	17.4–28.9	< 0.25	1.7–3.4	< 0.13	48.8–63.7	7.4–15.4	8.2–14.7
	Vitaclinical	17.2–23.4	< 0.26	1.3–2.6	< 0.24	48.6–64.5	11.5–18.9	3.8–8.2
	ZERAMEX	6.9–18.3	< 0.23	< 1.7	7.8–18.7	67.1–71.5	3.0–6.7	6.1–7.8
	Monobloc M10	4.6–28.0	< 0.40	< Det. limit	2.9–13.9	12.0–69.0	28.0–79.0	None

### Confocal laser scanning microscopy

**Fig. 4:**
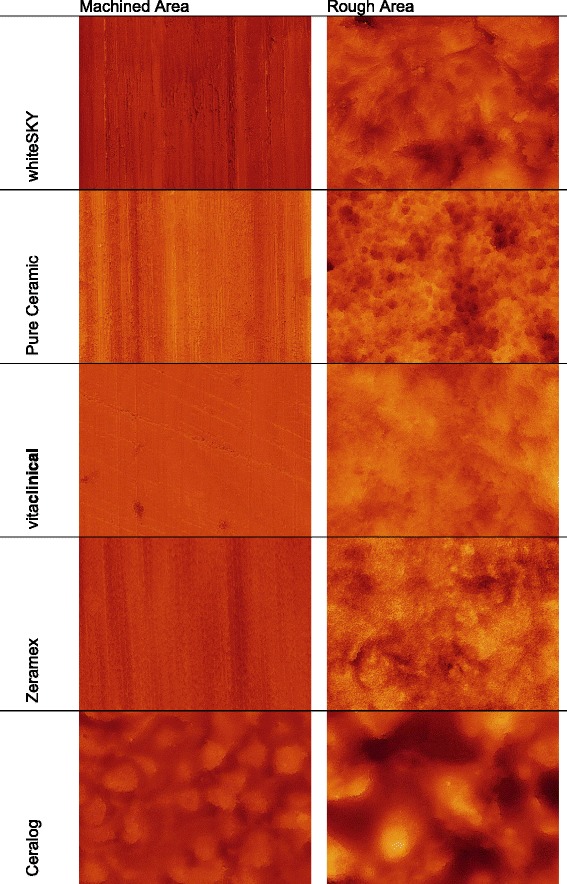
CLSM

Evaluation of the zirconia implant surface roughness as well as their surface texture parameters is carried out by means of confocal laser scanning microscopic technique. A Leica TCS SP2 (Leica Microsystems, Wetzlar, Germany) upright microscope with a red He-laser (633 nm) and a high-performance objective (HC PL FLUOTAR × 50/0.80) was used to acquire high spatial resolution images (1024 × 1024 pixels). Image stacks are created by capturing all the light reflected from the deepest to the highest point of the selected sample surface area. The image stacks are created in defined steps and acquired for five uniformly distributed points at the circumferences of representative-treated and none-treated locations on each type of implant (compare Fig. 4). The step size was calculated for optically optimized values by the LCS Leica confocal software. Because of the cylindrically shaped surface character, a zoom factor of 2 which generates an image size of 150 × 150 μm was used to avoid artificial height values.

**Fig. 5:**
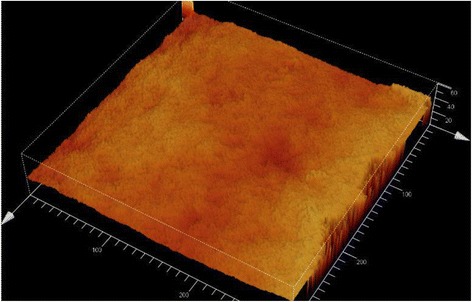
3D profile

Maximum projections and height distribution images (depth map) are calculated by LCS software from the image stacks and viewed exemplary in Fig. 5.

Subsequently, the depth map images are imported in the SPIP™ 4.2.6 (Image Metrology) software for roughness and texture evaluation. According to the ISO 25178-2 reference, all surface roughness parameters implemented in SPIP™ are evaluated and classified as amplitude, hybrid, functional, and spatial parameters. Selected values are shown in Table 3.

**Table 3:** Roughness analysis

		Amplitude parameters
Group	Name	*S*_a_ (μm)
Machined area	WhiteSKY	0.24 ± 0.04
	Straumann ZLA	0.36 ± 0.03
	Vitaclinical	0.20 ± 0.06
	ZERAMEX	0.30 ± 0.05
	Monobloc M10	0.61 ± 0.03
Rough area	WhiteSKY (Impl1)	0.91 ± 0.13
	Straumann ZLA (Impl2)	1.27 ± 0.24
	Vitaclinical (Impl3)	1.05 ± 0.17
	Zeramex (Impl4)	0.73 ± 0.95
	Monobloc M10 (impl5)	1.22 ± 0.36

## Results

### SEM

SEM micrographs presented in Fig. 2 demonstrate the dissimilarity of the sample surface microstructure. Implant 1 shows an overall smoother surface and a slaty-like surface without evidence of a typical etching process. The surface shows sparse roughness. Implants 2–4 show deep markings from their brand’s specific etching and sandblasting processes. In × 10,000 magnification, immersions can be found that look like little craters. Implant 2 shows the biggest immersions, and implant 4 shows the smallest. In a × 25,000 magnification, implants 2–5 show droplet-like-shaped particles on the outer surface as a basic structure of the immersions under × 10,000 magnification. The finest droplets can be found on implant 2, and the biggest droplets can be found on implants 4 and 5. Implant 5 stands out from the other implants. It shows very evenly spread droplets on the surface in every magnification (Fig. 2).

### EDX analysis

The semi-quantitative element composition showed no significant impurity of any implant tested (Table 2). Both the machined and the rough areas (Fig. 3) were predominated by zirconium, oxygen, and carbon. Yttrium could be found in implants 1–3. Implants 4 and 5 showed yttrium under the detection limit and just less than 1.7 atomic % in the apical aspect of implant 4. Minor traces of hafnium could be shown in all implants 1–5. Implants 1, 4, and 5 showed traces of aluminum on the surface. The highest amount of aluminum could be found on the surface of implant 4.

### Confocal laser scanning microscopy (CLSM)

CLSM images including the topological information of all five implants are shown in Fig. 4.

Untreated areas (machined areas) of implants 1–4 showed parallel grooves of the machining process in the interface area of the neck (Fig. 4). Treated areas (rough areas) show roughened surfaces due to special treatment with acid and sandblasting. Implant 5 showed roughened surface in both areas and no sign for a machined neck part.

### Roughness analysis (SPIP)

**Fig. 6:**
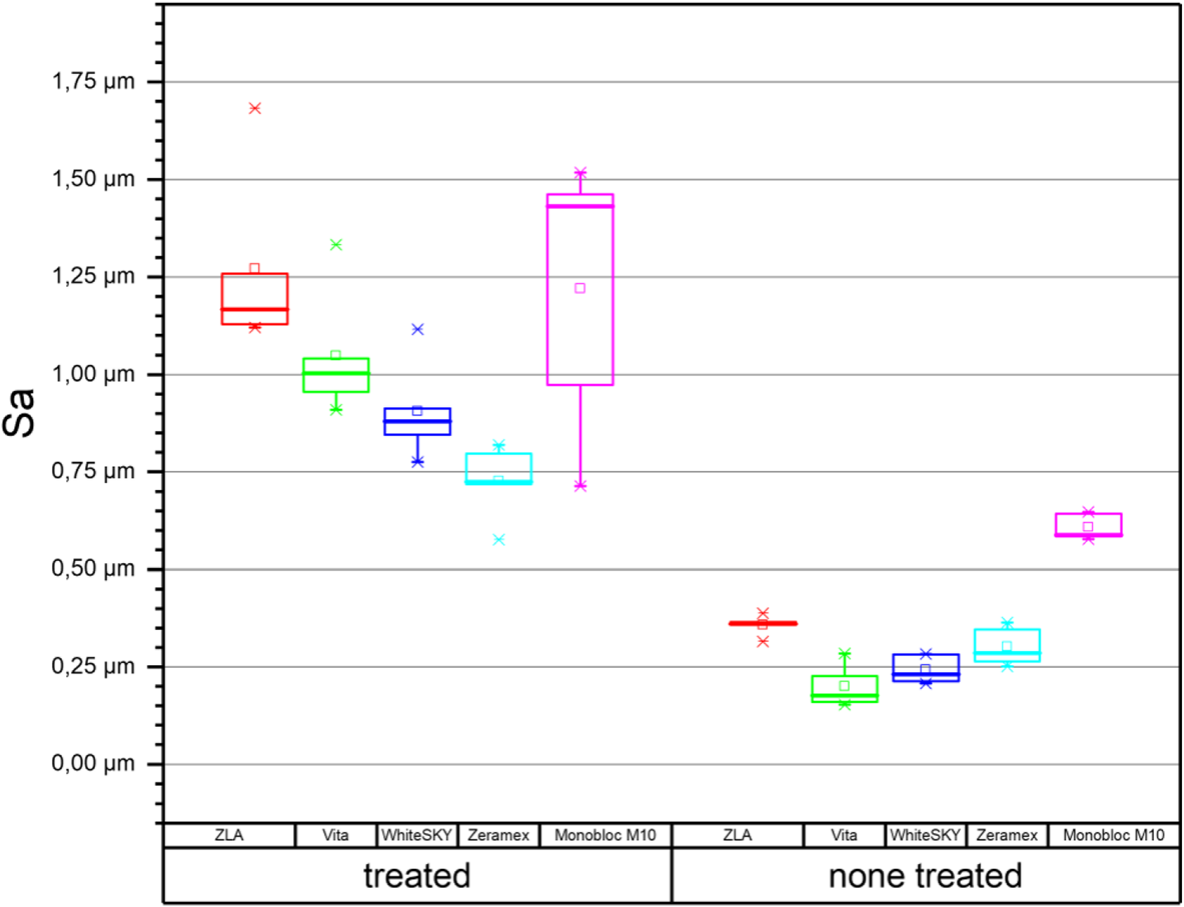
Roughness (*S*_a_) box plot

Implant 2 (*S*_a_ 1.27 μm ± 0.24) and implant 5 (*S*_a_ 1.22 μm ± 0.36) show the highest roughness values (*S*_a_) of all tested implants: Straumann’s pure ceramic implant was blasted and etched and shows the overall highest *S*_a_ value in the rough area. Implant 3 (vitaclinical) shows correspondingly lower *S*_a_ around 1.05 μm (± 0.17) (Table 3). The lowest *S*_a_ value could be found in implant 4, which was only sandblasted due to manufacturer’s specifications. However, the Zeramex implant despite being sandblasted and etched shows the lowest roughness value around 0.73 μm (± 0.95). Nevertheless, Zeramex shows a fine distribution of small pores all over the surface in the SEM sample images. Camlog’s Ceralog shows the highest roughness in the untreated area with 0.61 μm (± 0.03). Figure 6 shows the box plot of the roughness analysis with implant 5 having the widest distribution of measured values.

## Discussion

Implant surface characteristics are of ongoing scientific interest. Implants made from titanium are still the most common to be used. Titanium implants are made from alpha-beta alloy which consists of 6% aluminum and 4% vanadium (Ti-6Al-4V). These materials have low density, high strength, and resistance to fatigue and corrosion, and their modulus of elasticity is closer to the bone than any other implant material [18]. However, titanium implants are discussed to trigger hypersensitivity reactions due to surface corrosion [1, 19]. Titanium implant surfaces are machined, etched, sandblasted, and sometimes coated with special (company-specific) coatings. For titanium implants, roughness values (Ra) around 1.5 μm are known to provide successful osseointegration [20].

Ceramic implants experienced a renaissance since their reentry into the market. New ceramic implants with yttria (Y_2_O_3_)-stabilized tetragonal zirconium polycrystalline (Y-TZP) material have superior corrosion and wear resistance in comparison to titanium implants as well as high flexural strength (800 to 1000 MPa) [18]. However, due to manufacturing imperfections or flaws created during zirconia implant fabrication and because of special surface treatments, their strength can be compromised [18, 21]. Due to their brittle nature, ceramic implants tend to fracture. Especially sharp, deep, and thin threads can easily lead to implant failures [18, 21]. The surface treatment on ceramic is developed due to a process of sandblasting, etching, and heat treatment [22]. Sandblasting is usually done with alumina particles that lead to sharp edges and scratches on the surface. The treatment with hydrofluoric acid as the following procedure may smoothen the surface again [22–24]. However, in zirconia implants, due to stress caused by sandblasting, a tetragonal to monoclinic phase transition may be caused [22, 25]. This monoclinic volume fraction can be seen in 10–15% of the cases [26] and initially leads to a surface compression of the zirconia material [22]. According to Fischer et al., the long-term effects and the implant stability after this procedure are not yet proven [22]. However, it can be reversed by a thermal treatment that is higher than the transition temperature [22, 27].

The surface shape (droplet-like surface), which was observed in the SEM samples, can be caused due to the sintering process in which ceramic powder was melted and then formed. Different particle, immersion, and droplet sizes can also change due to possible reasons like usage of various types and dosages of acid for the etching process and change of exposure time to acid effect. A longer exposure time to etching process could also be responsible for lowering aluminum corundum from sandblasting processes. However, despite a very fine surface microstructure, implant 4 shows the highest amount of aluminum on the outer surface. This could be explained by sandblasting with aluminum-containing corundum particles followed by a shorter etching process. The higher amount of aluminum in implants 1, 4, and 5 might be due to the individual material composition while sintering the material mixture or to corundum particles of the machining and sandblasting process. Implants with aluminum under the detection limit could be caused by a final etching process. Implants 1 and 5 are not advertised with a special etching process. However, implant 4 is supposed to be etched. The etching could have happened before sandblasting, or the acid used was not strong enough to eliminate all aluminum particles.

All implants excluding the Ceralog Monobloc (implant 5) show typical parallel grooves of the machining process in the confocal laser scan and rougher surfaces in the treated areas. Ceralog is the only implant with a rough surface that can also be found in the machined area. Zirconia implants which are treated with a process of sandblasting, etching, and heat treatment are showing a micro-structured surface resulting in a surface roughness in the range of 1.2 μm [22]. In this study, implants 2 and 5 showed roughness values in the range of 1.2 μm. The other implants showed different roughness values. The surface porosity of titanium implants after sandblasting and etching processes is much more rigorous than that of the ceramic implants that were investigated. In this study, implants 2 and 5 can approximately be compared to titanium surface characteristics in the SEM samples. However, implant 5 was not sandblasted and etched because of a special “injection molding technique” and shows a wide distribution of roughness values. A similarity to the surface structure of titanium implants cannot be proven yet.

The semi-quantitative energy-dispersive X-ray spectroscopy (EDX) can be used to further analyze the components of the implant surface. None of the implants showed any impurity or unexpected results. Implants 4 and 5 showed yttrium under the detection limit in the EDX analysis. This could be caused by the lower dosage of yttrium endowment in the stabilization processing in comparison to other implants [17].

This investigation shows results on a sample basis with one implant tested and shall not be used for generalization.

## Conclusions

New ceramic implants are showing a variety of surface characteristics due to different manufacturing processes as shown by other groups [2, 28]. The surface structures of the investigated implants are close to titanium implants. If the surface characteristics really have a high influence on osseointegration, ceramic implants cannot yet compare to the long experience with titanium. However, there are several indications for using ceramic implants. In the future, ceramic implants have to prove themselves in clinical practice and clinical controlled trials.

## Abbreviations

AL_2_O_3_: Aluminum oxide
CIM: Ceramic injection molding
CLSM: Confocal laser scanning microscopy
EDX: Energy-dispersive X-ray spectroscopy
HIP: Hot isostatic pressing
I: Implant
kV: Kilovolt
mbar: Millibar
MPa: Megapascal
nm: Nanometer
*S*_a_: Area roughness parameter
SEM: Scanning electron microscopy
SLA™: Sandblasted, Large-grit, Acid-etched
Y-TZP: Yttrium-stabilized tetragonal zirconium polycrystalline
μm: Micrometer
